# Intriguing spin–lattice interactions in EuTiO_3_

**DOI:** 10.1038/s41598-021-98503-w

**Published:** 2021-09-23

**Authors:** Annette Bussmann-Holder, Efthymios Liarokapis, Krystian Roleder

**Affiliations:** 1 Max-Planck-Institute for Solid State Research, Heisenbergstr.1, 70569 Stuttgart, Germany; 2 Department of Physics, National Technical University of Athens, 15780 Athens, Greece; 3 Institute of Physics, University of Silesia, ul. 75 Pułku Piechoty 1, 41-500 Chorzów, Poland

**Keywords:** Physics, Condensed-matter physics, Ferroelectrics and multiferroics

## Abstract

During the last decade the cubic perovskite oxide EuTiO_3_ (ETO) has attracted enormous novel research activities due to possible multiferroicity, hidden magnetism far above its Néel temperature at T_N_ = 5.5 K, structural instability at T_S_ = 282 K, possible application as magneto-electric optic device, and strong spin–lattice coupling. Here we address a novel highlight of this compound by showing that well below T_S_ a further structural phase transition occurs below 210 K without the application of an external magnetic field, and by questioning the assumed tetragonal symmetry of the structure below T_S_ where tiny deviations from true tetragonality are observed by birefringence and XRD measurements. It is suggested that the competition in the second nearest neighbor spin–spin interaction modulated by the lattice dynamics is at the origin of these new observations.

## Introduction

Perovskite oxides are well known for their tendency to undergo various structural instabilities with multiple ground states ranging from ferroelectric, antiferroelectric, ferromagnetic, antiferromagnetic, skyrmion type, and complex combinations of those. This variability is the origin for their large application potential and constantly increasing research interests. While many of these compounds have been discovered in the early 1950’th^1^, the largest research activities were devoted to few of them only, e.g. PbTiO_3_ and homologues, where the unusual dielectric, pyroelectric, ferroelastic and polar features are promising properties for all types of applications. EuTiO_3_ (ETO) also belongs to this material class^2^, but has long been ignored by the research society since it was believed to be rather *boring* and only antiferromagnetism (AFM) has been reported below 5.5 K^3^. During the last 10 years the situation for ETO has changed considerably since the onset of AFM order has been shown to be correlated with an appreciable anomaly in the dielectric permittivity^4^. This—in turn—has been related to a soft optic mode reminiscent of a ferroelectric instability^5,6^ thereby suggesting the possibility of multiferroic capabilities. The alikeness of ETO with SrTiO_3_ (STO), namely almost the same lattice constants, cubic at high temperature, valency of Eu, Sr being + 2, empty Ti d-shell, has been pursued further and shown to be continued by the fact that ETO also undergoes a cubic to “tetragonal” phase transition^7–11^, however at much enhanced temperature as compared to STO^12–14^. The origin of this enhancement stems from strong spin–lattice coupling which on the one hand modifies the lattice dynamics, on the other hand the spin activity^7,15^. The interplay between both was predicted to induce “hidden” spin order visible in the magnetic susceptibility^16^, the birefringence^17^, the Raman response^18^, the lattice constants^19^ and the dielectric properties^20^. Very convincing breakthrough confirmations of these predictions have been achieved by muon spin rotation (µSR) experiments^21–24^, which are the most sensitive tools to detect any kind of magnetic order, even if this is not visible by bulk probing experiments. From birefringence as well as XRD, dielectric, and µSR data taken under the action of an external magnetic field^17,19–21^ a novel phase transition could be detected in ETO which takes place around 210 K and must be accompanied by a symmetry lowering. Since these experiments have been performed in an external magnetic field one could argue that the field causes this new phase which consequently should be absent once the field is removed. Intuitively, this assumption is supported by the strong spin–lattice interaction which is enhanced by a magnetic field. Also, high resolution XRD data could not detect any further transition except the one at T_S_. Here we show unambiguously by new birefringence data that the transition is also present *without* the field and even followed by another one at lower temperature. In addition, earlier XRD data^19^ together with the present birefringence data are explored in deeper detail and shown to exhibit features which are incompatible with true tetragonality. Our results may also apply to other related perovskites where the obvious and as-given taken structures hide tiny deviations from it and thus converge with results obtained from pair distribution function (PDF) data.

## Experimental

### Birefringence results

The birefringence data have been taken from an ETO film of thickness 1 µm grown on STO where in situ XRD experiments have confirmed the cubic structure at room temperature. Effects of strain can be excluded to be present since the films have the same lattice constant as the bulk material and in addition, ETO and STO have almost identical lattice constants. Also**,** the transition to antiferromagnetic order has been verified at T_N_ = 5.5 K. Furthermore the structural phase transition at T_S_ = 282 K was confirmed by XRD measurements and birefringence. Therefore, we conclude that the films are indeed ETO and no signatures of any other phase are present.

Details of the experimental technique have been described in^25^. In that paper, domain structures in the investigated ETO film were observed for which the birefringence value Δn has been derived. This structure was well visible after subtracting from each orientation map at given temperature the “background” map referring to the one taken at 320 K, i.e. far enough away from the structural phase transition temperature T_S_ = 282 K and well in the cubic phase. Based on this map we were able to calculate the birefringence for regions corresponding to a well-defined indicatrix orientation. Thus, it is possible to conclude from such data about a potential phase transition. This can be rather easily visualized through a color code, which corresponds to different indicatrix orientations.

**Figure 1:**
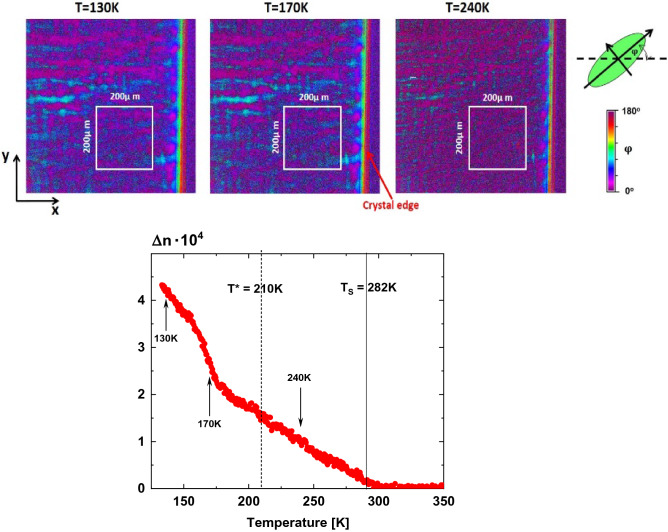
(Lower Part) Temperature dependence of Δn(T) of ETO. The linear regime of the temperature dependence, as expected from, e.g., Landau theory, is observed below T_S_. The full vertical line at 282 K corresponds to the onset of symmetry lowering at T_S_. A dashed line is placed at the point where transition near 210 K is suggested, although conclusive evidence is discussed further in the text (see Fig. 2). (Upper Part) there are exemplary birefringence maps of ETO taken at temperatures T = 130 K (left), T = 170 K (middle), and T = 240 K (right). The white framed squares of size 200 µm × 200 µm are those regions for which Δn(T) has been determined. The colour code indicates the indicatrix angle φ.

Before going into more details with respect to the indicatrix orientation, the temperature dependence of the birefringence of ETO, as reported by us earlier, is shown in Fig. 1. This dependence has been plotted based on calculations for the regions of the size 200 µm × 200 µm. There are apparent, though much smaller than in a magnetic field, signatures of several phase transitions. As expected, the birefringence Δn is zero for T > T_S_, i.e. in cubic symmetry. Slightly above T_S_ a small enhancement is visible in accordance with precursor effects which have been observed in many perovskite oxides^26^. Below T_S_ Δn increases linearly with decreasing temperature and follows a Landau type behavior which can be fitted by Δn ~ (T_S_–T)^β^, β = 1^26^, up to approximately T* ~ 210 K. Below this temperature, the data are no more describable by this law but a clear new upturn appears which seems to be again linear in temperature with a critical exponent *β* ⁓ 1/2. We can definitely exclude any effects from the substrate since the STO related birefringence is not only much smaller than the one of ETO but is also zero above 105 K where the cubic to tetragonal phase transition of STO takes place.

In order to define the structural changes related to T_S_ and T*, the maps of indicatrix orientations in Fig. 1 were analyzed in detail. Around 240 K, i.e. in the tetragonal phase, the crystal is almost monochrome as would be anticipated in tetragonal symmetry. However, it is important to note that some strikes are appearing which might not be compatible with genuinely tetragonal symmetry. At 170 K and below a clear stripe-like structure is visible and a multicolored impression appears. From the color code this can be identified as stemming mainly from zero and 90° indicatrix, as could be in the tetragonal phase. However, this directly demonstrates that the symmetry is lower than tetragonal.

**Figure 2:**
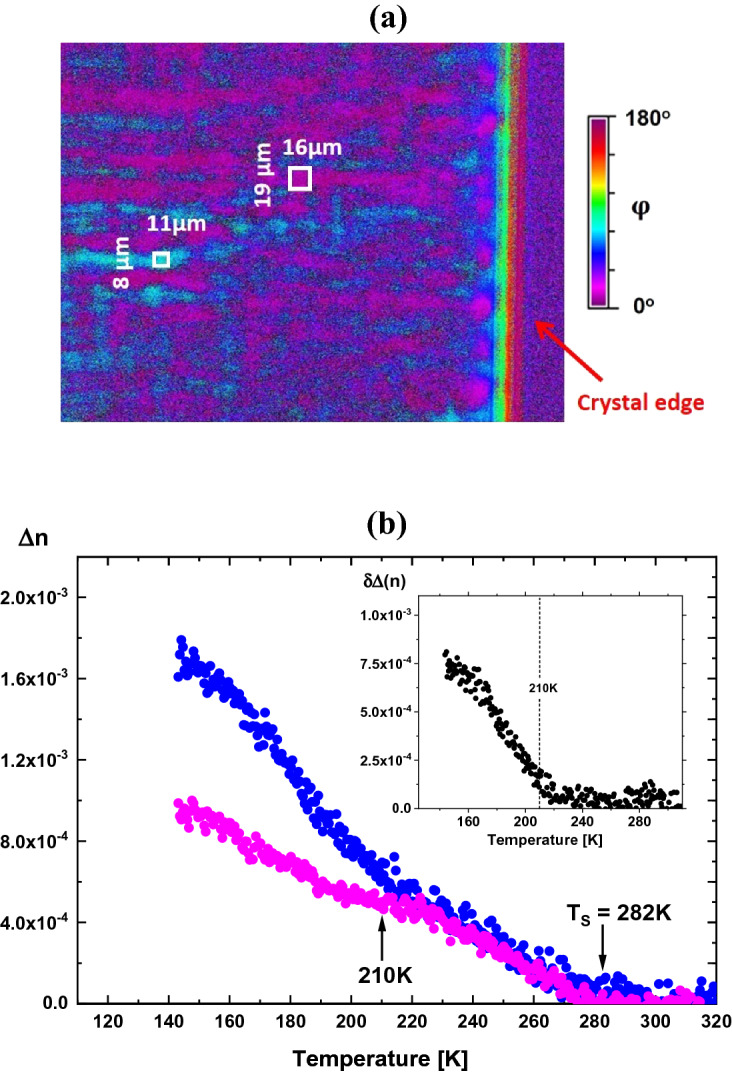
(**a**) Example of indicatrix orientations at temperature T = 140 K. Two types of indicatrix orientations corresponding to [100] (magenta) and [010] (blue) can be distinguished. (**b**) Temperature dependence of the birefringence calculated for small regions marked by the white framed rectangular in (**a**). Below this temperature, the abrupt change of Δn is a signature of a different phase transition. The inset in Fig. 2b is the difference in birefringence between the [100] and [010] orientations. While the transition near 210 K is well visible, slightly above this temperature, some traces of the Δn might be incompatible with the tetragonal symmetry.

Assuming that the symmetry for T < T_S_ is tetragonal, the Δn indicates the intrinsic value measured in the [100] direction. The setup used for the birefringence measurement detects absolute values |Δn|. Thus, in the case of the tetragonal symmetry, we should obtain the same birefringence value with the slow axis oriented 0° and 90° to the [100] direction. However, it is shown in Fig. 2 that for T > 210 K, the Δn(T) runs are slightly different.

The birefringence changes below 210 K, calculated for a region of the size 200 µm × 200 µm, provide a very pronounced difference in the two temperature runs typical for a symmetry change and as expected for another structural phase transition. It might be suspected that the size of the region used for the calculations is too large, mirroring an average effective value of Δn. In order to exclude any artefact like this, two regions with smaller size have been selected lying within domains along of [100] and [001] orientation. The Δn(T) values for these regions are shown in Fig. 2. While for T < T_S_, these two dependencies are almost identical (see inset to Fig. 2b) below T⁓220 K, they distinctly split into two independent runs. This is a clear signature of another phase transition in ETO to a symmetry lower than tetragonal.

**Figure 3:**
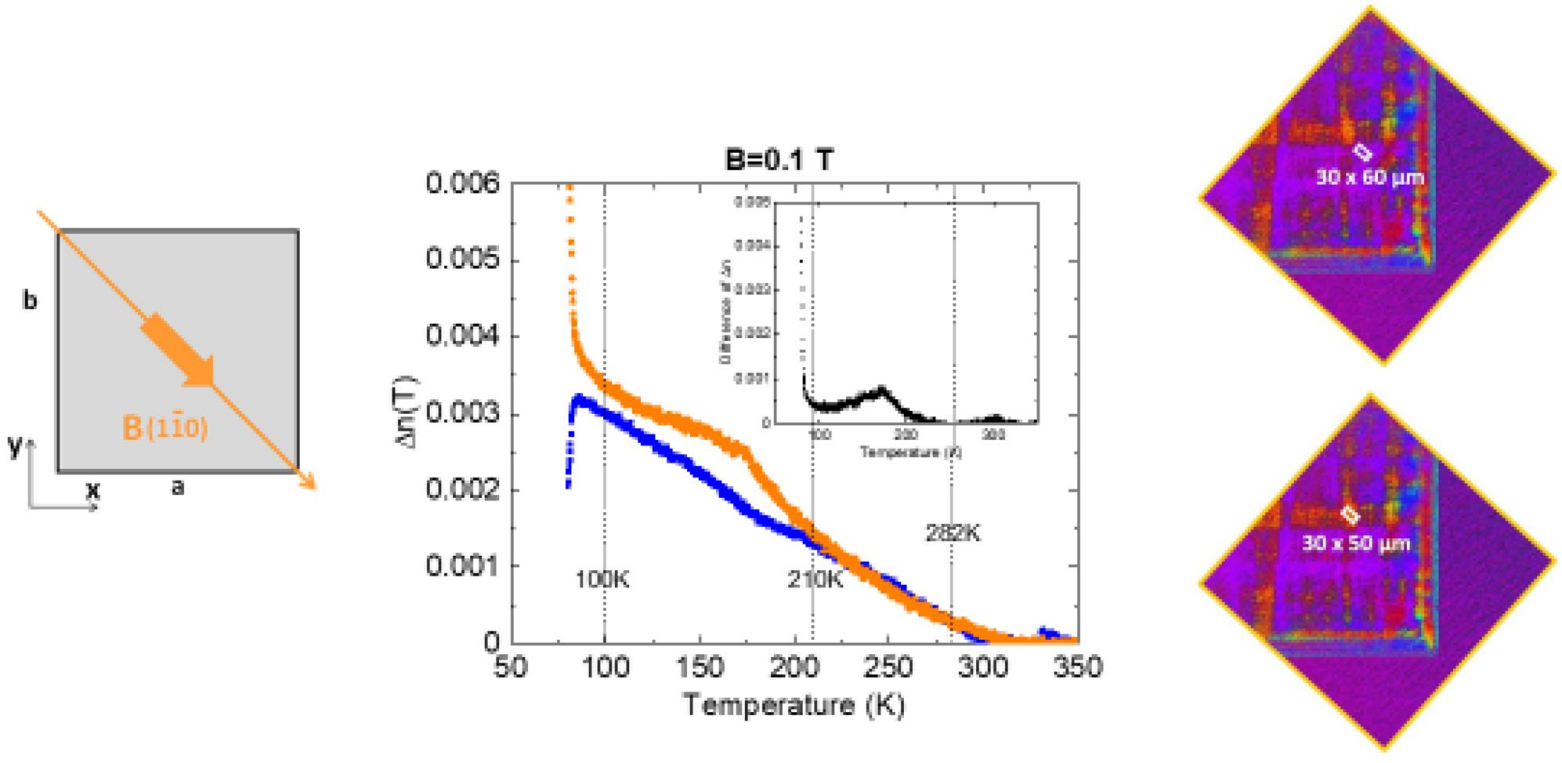
Temperature dependence of Δn(T) in an applied magnetic field of 0.1 T with the field orientation as shown on the left side of the figure. Δn has been calculated for the white framed squares shown on the right side of the figure. The black symbols are the difference between the blue and the orange data.

In various recent papers, we have reported that bulk ETO and films of ETO are susceptible to an external magnetic field of relatively low intensity. Hence, we have also carried out calculations for two small regions (domains) for our film sample that correspond to two different crystallographic orientations (blue and orange). As is shown in Fig. 3, a magnetic field of H = 0.1 T has the following effects:

- It increases the value of Δn(T), but it does not destroy tetragonal phase below T_S_,
- It splits Δn(T) into two different temperature dependencies below T = 210 K, corresponding to a symmetry lowering,
- Below T = 100 K the temperature dependence of Δn changes dramatically and points to another phase transition at this temperature.
- Most striking is the fact that the magnetic field changes the character of the split of the Δn(T) than that detected in the absence of the field (Fig. 2). It once again points to another phase transition below 210 K. The two Δn(T) runs above 210 K suggest that the magnetic field stabilizes the tetragonal symmetry of the lattice and even extends it beyond T_S_.

### XRD data

**Figure 4:**
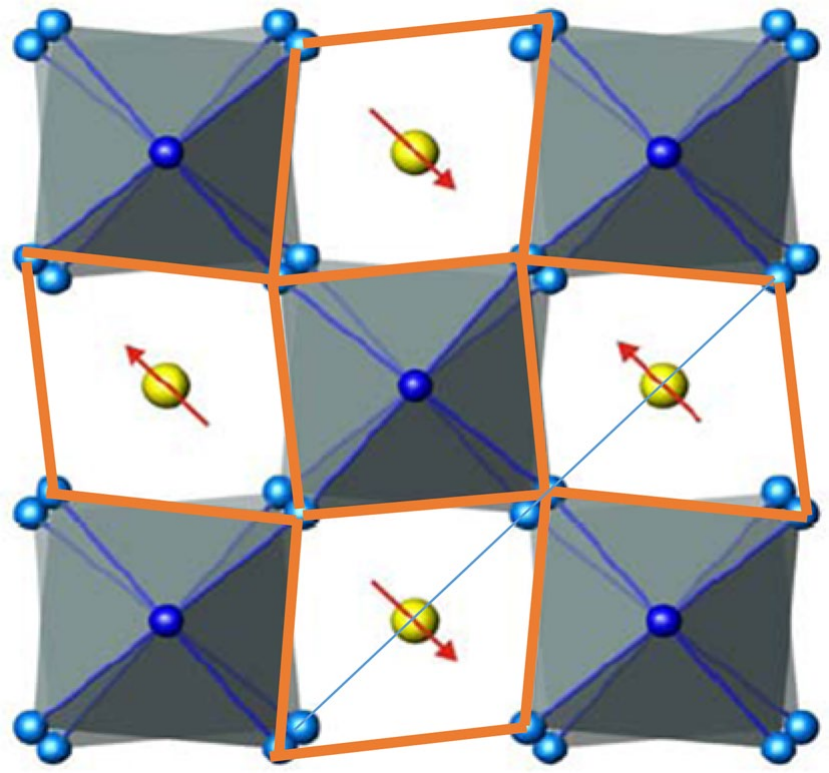
Schematic structure of the tetragonal phase of ETO. The grey shaded areas correspond to the TiO_6_ octahedra, the yellow circles represent the Eu atoms with the arrows assigned to the spin orientation in the AFM phase. The orange lines highlight the tetragonal distortion.

The doubts about the tetragonality below T_S_ are further supported by very recent XRD measurements^19^ performed at the XRD1 beamline of Elettra Synchrotron (Trieste), where detailed data for the Eu_O distances in the “tetragonal” phase have been taken, assuming the tetragonal (I4/mcm space group) for the lower than room temperature data. Since in this phase three Eu_O bond distances are realized (see Fig. 4), namely the apical one and two planar ones, the planar ones must follow the relation [Eu_O_2A_(T)] + x = [Eu_O_2B_(T)]-x where Eu_O_2A_ is the shorter distance oxygen atom and Eu_O_2B_ the longer one and x is the bond length shift caused by the tetragonal distortion. Eu_O_1_ refers to the apical oxygen Eu_O bond length. Upon subtracting from all the bonds the corresponding temperature dependent average Eu_O_average_ of the three measured distances at the respective temperature T, i.e. [Eu_O_2A_(T) + Eu_O_2B_(T) + Eu_O_1_(T)]/3 = Eu_O_average_(T), the difference values: Eu_O_2A_(T)-Eu_O_average_(T) = Δ_2A_(T); Eu_O_2B_(T)-Eu_O_average_(T) = Δ_2B_(T), Eu_O_1_(T)-Eu_O_average_(T) = Δ_1_(T) are obtained; all of them should approach continuously zero when approaching T_S_ from lower temperatures. In addition, the two planar distances, namely Δ_2A_(T) + Δ_2B_(T) should add up to zero for all temperatures below 282 K, as well as the difference (Eu_O_2A_ + Eu_O_2B_)/2-Eu_O_1_.

**Figure 5:**
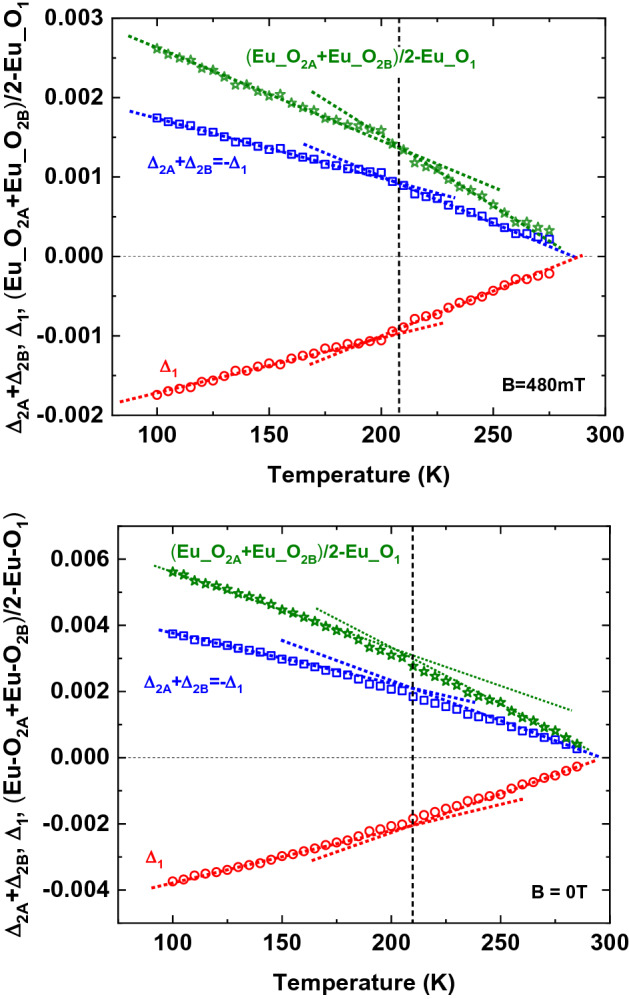
Bond distance variations from the average of the sum of the planar Δ_2A_ + Δ_2B_ distance deviations (blue squares), the apical one Δ_1_ (red circles), and half of the sum of the two planar distances minus the apical one (green stars) in zero field (left) and in a field of 480mT (same symbols and colors). Bond length values were obtained from Rietveld analysis assuming the tetragonal I4/mcm space group. The crossing of the dotted lines (same color code as the symbols) indicates the temperatures where the gradient of the linear regions changes. The dashed black vertical lines are a guide to the eye for the temperature where the mentioned anomalies occur.

However, neither the difference sum nor the apical Eu_O_1_ difference follows these considerations. (Fig. 5 left). Around T⁓210 K (dashed lines with colors corresponding to the respective symbols) all quantities show a break in the linear temperature dependence and adopt a new gradient. This temperature coincides approximately with the one where the birefringence data also show a new slope and a splitting of the domain structure (Figs. 2, 3). Upon applying a small magnetic field of 0.48 T all three values are decreased by more than a factor of 2, i.e., tetragonality is on the verge of being restored (Fig. 5 right). Amazingly, also under the action of the field again the temperature of ⁓210 K plays a borderline temperature where the inclination of the linearity changes even more pronouncedly as compared to zero field. These results together with the ones obtained from birefringence support the conclusion that the tetragonal phase is not truly tetragonal, but that small deviations from it are present which are re-established by an external magnetic field.

## Discussion

One might argue that the observed length differences (Fig. 5) are too tiny to influence any possible magnetic or structural responses, however, in various earlier work it has been shown that changes in the lattice constant, the Eu–O bond distance, respectively, of less than 0.01%, i.e. of the order of 10^–4^ Å, can cause, e.g., a ferroelectric phase transition or a transition from AFM to PM. Also, pair distribution function (PDF)^27–29^ data support these results. It implies that ETO is on the verge of various structural instabilities under pressure or strain^30^ where in^30^ support for deviations from tetragonality have been observed at low pressure.

The spin Hamiltonian in the antiferromagnetic phase of ETO reads:

1 $$H={z}_{1}{\sum }_{\left[i,j\right]}{J}_{1}{S}_{i}{S}_{j}+{z}_{2}{\sum }_{\langle i,j\rangle }{[J}_{2}+\alpha \langle {{W}^{2}}_{T}\rangle {]S}_{i}{S}_{j}-\mathrm{h}{\sum }_{i}{S}_{i}$$

where $h={g}_{i}{\mu }_{B}{B}_{0}$, with ${g}_{i}$=gyromagnetic moment,${\mu }_{B}$=Bohr magneton, and ${B}_{0}$=external magnetic field. *J*_1_, *J*_2_ are the nearest and next nearest neighbor spin–spin interactions with *J*_1_/k_B_ =  − 0.021 K, *J*_*2*_/k_B_ = 0.040 K, *S*_i_ = 7/2^31^, *J*_1_, *J*_2_ are the nearest and next nearest neighbor spin–spin interactions, *α* is the spin–lattice coupling constant, *z*_1,2_ are the nearest and next nearest neighbors, and $\langle {{W}^{2}}_{T}\rangle$ is the self-consistently determined thermal average of the polarizability coordinate. In the paramagnetic and pseudo-tetragonal phase *J*_1_ is too small to contribute to any magnetic activity, whereas the second term *J*_2_ splits into three according to the tetragonal splitting of the Eu_O bond lengths, i.e. *J*_2, Eu-O1_, *J*_2, Eu-O2_, *J*_2, Eu-O2*_. The polarizability coordinate of ETO increases with temperature from 0.04 Å to a maximum 0.065 Å, where tiny changes in it have dramatic consequences for the lattice and spin dynamics. In a magnetic field $\langle {{W}^{2}}_{T}\rangle$ has to be replaced by:

2 $$\langle {{\tilde{W }}^{2}}_{T}\rangle =\langle {{W}^{2}}_{T}\rangle -{\Delta a\left(T,H\right)}^{2}$$

Since the bare *J*_2_ is also almost zero in the paramagnetic phase as *J*_1_, the next nearest neighbor spin–spin interaction is exclusively dominated by the polarizability coordinate, a dynamical quantity, and the sensitivity of the lattice constant to a magnetic field. Both are known from earlier work^22,32^. The magnetic field strength used in XRD and birefringence experiments is small (< 0.5 T), and in this external field the lattice constant increases nonlinearly. It has the consequence that the renormalized polarizability coordinate (Eq. 2) representing here the spin–spin exchange interaction, changes its sign by the tetragonal distortion**,** especially in the plane where one bond is increased while for the other the opposite happens. It means that for the Eu_O_2A_ bond the renormalized spin–spin interaction ${\tilde{J }}_{2}$ is attractive, i.e. AFM, whereas for Eu_O_2B_ it is repulsive, i.e. FM with ${\tilde{J }}_{2}$ being given by:

3 $${\tilde{J }}_{2}={[J}_{2}+\alpha \langle {{W}^{2}}_{T}\rangle {]S}_{i}{S}_{j}]$$

as long as the magnetic field is zero. With field it changes according to Eq. 1. This produces a highly frustrated situation for the spin state, which can be reverted by returning to the tetragonal phase. In addition, this frustration causes the deviations from the ideal tetragonal structure in order to minimize the spin induced energy increase. With decreasing temperature the lattice contracts further to reach the temperature where the spin frustration is lifted by a symmetry lowering to orthorhombic or even lower symmetry. Unfortunately, we are not able to either determine experimentally the true symmetry of the “nearly tetragonal” phase or the one of the phase below 210 K since the changes in the lattice constants are too small to be detected (Rietveld analysis assuming various possible space groups could not provide decisive conclusions). Also theoretically it is extremely difficult to make any predictions based on density functional theory (DFT)^33^ since the energy differences between the possible space groups are very small (a few meV, depending on the size of the Hubbard U term) to unambiguously assign the symmetry. However, in^33^ it was concluded that three candidate ground states:a^0^a^0^c^−^ (*I4/mcm*), a^0^b^−^b^−^ (*Imma*), and a^−^a^−^a^−^ (*R ¯3c*) are most likely realized where again the calculated energy differences are too small to allow the distinction which of these phases as the ground state. Even, a phase coexistence has been discussed. In another DFT study on ETO *I4/mcm*, *Imma*, *Pnma*, and *C2/c* have been identified as the lowest energy candidates^34^. Only from our earlier data taken in a magnetic field^17^ we find as possible and likely candidates *Cmcm* and *C2/m* or even lower symmetries. A detailed crystallographic study of the related compound EuNbO_3_^35^ where Eu also has the valency + 2, has identified two phase transitions where the cubic high temperature symmetry is lowered to tetragonal at T_S_ = 460 K and further lowered to orthorhombic *Imma* at T* = 360 K. The latter symmetry would be consistent with our birefringence data in a magnetic field. In^36^ the mixed crystal serie EuTi_1-x_Nb_x_O_3_ has been investigated by resonant ultrasound spectroscopy where a systematic increase in T_S_ with increasing x was observed, consistent with^35^ with the highest T_S_. Combining these two studies and including the present results suggests that the here reported structural transition at 210 K should also be present in the mixed crystal series and increase systematically with x to 360 K at x = 1.

## Summary

In summary, we have presented unambiguous experimental evidence for first a second structural phase transition below T_S_ to orthorhombic or even lower symmetry around 210 K. Second, both data sets, birefringence and XRD, reveal small deviations from true tetragonality, which cause a competition of the second nearest neighbor spin–spin interaction. This competition can be compensated by a magnetic field, which restores the tetragonal symmetry. All effects reported here can only be understood by intriguing spin–lattice interactions, which are interwoven to modify the lattice properties and the spin activity in an intricate way.
