# Author Correction: Intriguing spin–lattice interactions in EuTiO_3_

**DOI:** 10.1038/s41598-022-06487-y

**Published:** 2022-02-08

**Authors:** Annette Bussmann‑Holder, Efthymios Liarokapis, Krystian Roleder

**Affiliations:** 1 Max-Planck-Institute for Solid State Research, Heisenbergstr.1, 70569 Stuttgart, Germany; 2 Department of Physics, National Technical University of Athens, 15780 Athens, Greece; 3 Institute of Physics, University of Silesia, ul. 75 Pułku Piechoty 1, 41‑500 Chorzów, Poland

Correction to: *Scientific Reports* 10.1038/s41598-021-98503-w, published online 23 September 2021

The Funding section in the original version of this Article was incomplete:

“Open Access funding enabled and organized by Projekt DEAL.”

now reads:

“Open Access funding enabled and organized by Projekt DEAL. The synchrotron xrd research leading to this result has been supported by the project CALIPSO plus under Grant Agreement 730872 from the EU Framework Programme for Research and Innovation HORIZON 2020."

The original Article has been corrected.

